# Allergy to pomegranate and artichoke, novel food allergens of the Mediterranean diet

**DOI:** 10.1186/2045-7022-3-S3-P75

**Published:** 2013-07-25

**Authors:** L Macchia, LA Giliberti, A Lotti, M-P Rossi, G Kourtis, E Damiani, A Ferrannini, A Bellotti, L D'Elia, A Nico, M Vacca, MF Caiaffa

**Affiliations:** 1 Allergology and Clinical Immunology, University of Bari - Aldo Moro, Bari, Italy; 2 Allergology and Clinical Immunology, University of Foggia, Foggia, Italy

## Background

Epidemiology of food allergy is currently the focus of considerable research interest. Particularly important appears the recognition of novel food allergens and the characterization of specific food allergy patterns, relevant to homogeneous geographic areas with distinct alimentary habits, such as the Mediterranean area. Here we report on recent findings on allergy to Mediterranean fruits and vegetables.

### The *Punica granatum* (pomegranate) project

A handful of cases of hypersensitivity to this, otherwise valuable, Mediterranean fruit have been described. Three patients with multiple sensitizations to food allergens and an unequivocal history of hypersensitivity upon ingestion of pomegranate were identified. *P. granatum* specific IgE *in vivo* were detected by the prick by prick method with fresh pomegranate seeds. We then used a 100,000 x g supernatant of *P. granatum* homogenate, with a protein content of 0.8 mg/ml, for standard skin prick testing, eliciting skin responses twice as stronger, compared to the fresh fruit. Moreover, by combining ammonium sulphate precipitation, ultrafiltration and gel filtration, we biotinilated proteins with *M*_r_ > 10,000 and developed a RAST capture ELISA, which revealed the presence of IgE specific for *P. granatum* in the sera of the 3 patients (1.4 to 5.4 KU/ml). The assay proved negative in 4 out of 5 controls examined (one false positive).

### The *Cynara scolymus* (artichoke) project

A very limited number of cases of food allergy due to artichoke have been reported in the literature. Four patients with a convincing clinical history of adverse reaction following ingestion of artichoke were identified. The diagnosis was confirmed by prick by pricking with the fresh vegetable and standard skin prick tests with a 100,000 x g supernatant of artichoke homogenate. Moreover, by skin prick test and RAST all the patients tested positive to several *Compositae* pollen allergens. (Interestingly, *C. scolymus* also belongs to this family). By using the techniques mentioned above we were able to show the presence of IgE specific for *C. scolymus* in the sera of the patients studied. Finally, by RAST inhibition experiments, we demonstrated IgE cross-reactivity between the weed *Artemisia vulgaris* pollen and *C. scolymus*. Based on these results, we speculate that a food/pollen *Compositae* allergy may develop in people living in areas with a diet rich in edible *Compositae*, as the Mediterranean diet.

## Disclosure of interest

None declared.

